# Enhanced visualization of vessels in submucosa by amber red color imaging in colonic endoscopic submucosal dissection

**DOI:** 10.1055/a-2351-3985

**Published:** 2024-07-08

**Authors:** Ryohei Maruoka, Mitsuru Esaki, Yosuke Minoda, Yoshihiro Otsuka, Kazuhiro Haraguchi, Haruei Ogino, Eikichi Ihara

**Affiliations:** 1 Department of Medicine and Bioregulatory Science, Kyushu University, Graduate School of Medical Sciences, Fukuoka, Japan; 2 91356 Department of Gastroenterology, Harasanshin Hospital, Fukuoka, Japan; 3 Department of Metabolism and Gastroenterology, Kyushu University, Graduate School of Medical Sciences, Fukuoka, Japan

Intraoperative bleeding is a major complication of endoscopic submucosal dissection (ESD). Various devices and techniques have been developed to control intraoperative bleeding 1 2 3 4 . Amber red color imaging is a recently developed observation modality that integrates brightness and color-enhancement technology used in linked color imaging, characterized by an increased proportion of long-wavelength relative linked color imaging, which targets the mucosa 5 . These amber red color imaging features improve the visibility of blood vessels and active bleeding points in the deep submucosa. Herein, we present a case of a colonic laterally spreading tumor that was treated with ESD using amber red color imaging ( Video 1 ).

Enhanced visualization of vessels in submucosa by amber red color imaging in colonic endoscopic submucosal dissection.Video 1

A 25-mm laterally-spreading tumor was detected in the sigmoid colon. ESD was performed using an electrosurgical knife (Flush Knife BT 1.5; Fujifilm, Tokyo, Japan) and EC-760ZP-V/M colonoscope (Fujifilm) paired with an ELUXEO 7000 video system (Fujifilm). Mucosal injection and circumferential incision followed by submucosal dissection were performed from the anal side to the oral side. With the blue color of the submucosa preserved, the overall visibility of the submucosa obtained with amber red color imaging was equivalent to that obtained with white light imaging (WLI). The visibility of vessels in the deep submucosa is greatly enhanced by amber red color imaging, which facilitates the easy and safe precoagulation of thick vessels and reduces the risk of intraoperative bleeding ( Fig. 1 ). Even when intraoperative bleeding occurred in the submucosa, amber red color imaging visualized active bleeding points as deep yellow against a background of surrounding yellow, indicating accumulated blood, whereas these points were not clearly visible with WLI ( Fig. 2 ). Using amber red color imaging, quick and effective hemostasis was achieved in this case and en bloc resection was achieved without complications.

**Fig. 1:**
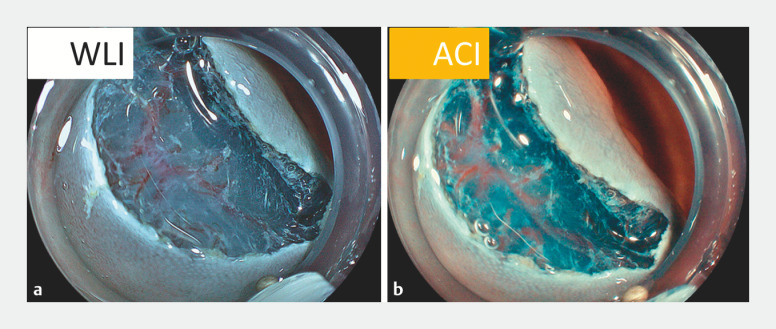
Endoscopic images during mucosal incision obtained with white light imaging and amber red color imaging. **a** White light imaging. **b** Amber red color imaging.

**Fig. 2:**
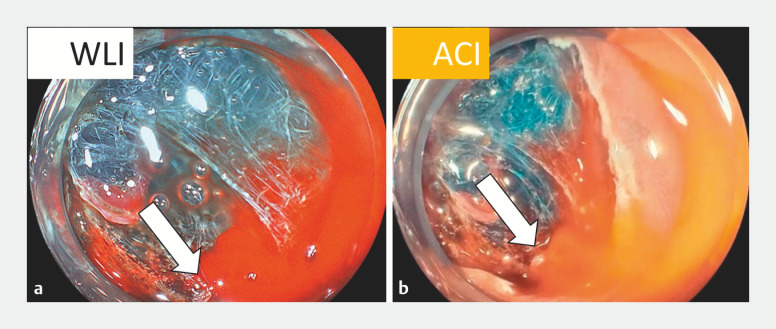
Endoscopic images of active bleeding during submucosal dissection obtained with white light imaging and amber red color imaging. Arrow indicates the active bleeding point. **a** White light imaging. **b** Amber red color imaging.

In conclusion, amber red color imaging in ESD could be useful for reducing the risk of intraoperative bleeding by identifying thick vessels and active bleeding points, facilitating quick and safe hemostasis. Amber red color imaging makes ESD easier and safer.

Endoscopy_UCTN_Code_TTT_1AQ_2AD_3AD

## Notes

Endoscopy E-Videos https://eref.thieme.de/e-videos
*E-Videos* is an open access online section of the journal *Endoscopy* , reporting on interesting cases and new techniques in gastroenterological endoscopy. All papers include a high-quality video and are published with a Creative Commons CC-BY license. Endoscopy E-Videos qualify for HINARI discounts and waivers and eligibility is automatically checked during the submission process. We grant 100% waivers to articles whose corresponding authors are based in Group A countries and 50% waivers to those who are based in Group B countries as classified by Research4Life (see: https://www.research4life.org/access/eligibility/ ). This section has its own submission website at https://mc.manuscriptcentral.com/e-videos .

## References

[1] Kita A Kuribayashi S Itoi Y Efficacy of using red dichromatic imaging throughout endoscopic submucosal dissection procedure Surg Endosc 2023 37 503 509 10.1007/s00464-022-09543-w 36001152

[2] Ikenoyama Y Iri A Imai H Endoscopic submucosal dissection of an early-stage gastric tumor with minimal bleeding using full-time red dichromatic imaging Endoscopy 2022 54 E1001 E1002 10.1055/a-1887-6024 35926527 PMC9736812

[3] Tanaka S Toyonaga T Morita Y Efficacy of a new hemostatic forceps during gastric endoscopic submucosal dissection: A prospective randomized controlled trial J Gastroenterol Hepatol 2017 32 846 851 10.1111/jgh.13599 27648821

[4] Maehara K Esaki M Sumida Y Comparison of hemostatic ability between spray coagulation and forced coagulation modes in endoscopic submucosal dissection in patients with early gastric neoplasms: a study protocol for multicenter randomized controlled trial (Spray-G trial) Trials 2024 25 53 10.1186/s13063-023-07852-6 38225659 PMC10788983

[5] Shinozaki S Kobayashi Y Hayashi Y Colon polyp detection using linked color imaging compared to white light imaging: Systematic review and meta-analysis Dig Endosc 2020 32 874 881 10.1111/den.13613 31869487

